# Improvement of Interfacial Adhesion and Thermomechanical Properties of PLA Based Composites with Wheat/Rice Bran

**DOI:** 10.3390/polym14163389

**Published:** 2022-08-19

**Authors:** Vito Gigante, Laura Aliotta, Ilaria Canesi, Marco Sandroni, Andrea Lazzeri, Maria-Beatrice Coltelli, Patrizia Cinelli

**Affiliations:** 1 Department of Civil and Industrial Engineering, University of Pisa, 56122 Pisa, Italy; 2 INSTM—Inter University Consortium of Material Science and Technology, 50121 Firenze, Italy; 3 Planet Bioplastics s.r.l., 56127 Pisa, Italy

**Keywords:** biobased waxes, natural fibers, mineral fillers

## Abstract

The present work aims to enhance the use of agricultural byproducts for the production of bio-composites by melt extrusion. It is well known that in the production of such bio-composites, the weak point is the filler-matrix interface, for this reason the adhesion between a polylactic acid (PLA)/poly(butylene succinate)(PBSA) blend and rice and wheat bran platelets was enhanced by a treatment method applied on the fillers using a suitable beeswax. Moreover, the coupling action of beeswax and inorganic fillers (such as talc and calcium carbonate) were investigated to improve the thermo-mechanical properties of the final composites. Through rheological (MFI), morphological (SEM), thermal (TGA, DSC), mechanical (Tensile, Impact), thermomechanical (HDT) characterizations and the application of analytical models, the optimum among the tested formulations was then selected.

## 1. Introduction

Agriculture generates large quantities of organic residues. An incorrect handling of these by-products undermines its environmental sustainability. Thus, the development of pioneering processes for the use of this “waste products” is necessary to implement the principles of the circular economy [1,2]. Agricultural residues may derive from the production flow or from subsequent processing operations. As for the former, they consist mainly of stalks and leaves, while the by-products of processing operations are typically the remaining parts of the grain, and these components will be the focus of this paper [3,4]. These types of biomass can reach enormous quantities, estimated at 150 Mtons/year [5], which are basically used in the feed industry, and their disposal can cause environmental or health damage. For this reason, further utilization of these residues is a goal that must be pursued in order to reduce the wastes ang giving them an added value [6]. In this framework the wide availability of wheat bran and rice bran residues, derived from processing byproducts of cereal milling factories, can be used for producing biodegradable composites, in fact the production of biobased and/or biodegradable items with natural fillers embedded in a biopolymeric matrix can be a valid processing option to guarantee a second life to byproducts of this type [7,8]. Currently, wheat and rice bran are “recycled” as feeds at a very cheap price [9] and many technological steps ahead must be carried out to achieve value-added manufactured goods using bran derivates [10,11,12]. Indeed, the production of biocomposites must take into account difficulties to be overcome and faults to be avoided, at this purpose Sasimovski et al. [13] carried out a detailed study regarding the addition of wheat bran to a biopolymeric matrix through a twin screw extruder, noticed that modifying the extruder screw rotational speed caused a noteworthy impact on both the technology efficacy and products final properties. For the achievement of a biocomposite with desired properties, it is fundamental to guarantee an homogeneous dispersion of the filler into the polymeric matrix [14]. Nonetheless, in contrast to the hydrophobic characteristic of many polymer matrices, the typical hydrophilicity of fibers can cause water absorption cracks and consequently reduce the strength of the composite. [15]. The weak compatibility of fiber/matrix coupling can be increased by the treatment of the fiber surface through chemical or physical methods [16], for this reason, many researchers are working to find the tailored treatment that can improve the adhesion of peculiar systems of natural fillers and biopolymer matrices [17]. In this context the present paper aims to improve the interfacial adhesion between a blend of bio-polyesters and wheat/rice bran through the use of a treatment based on a biobased wax. More specifically the co-continuous blends used in this work is constituted by 60 wt.% of Poly(lactic acid) (PLA) and 40 wt.% of poly(butylene succinate-co-adipate) (PBSA) and it has been selected as matrix for the dispersion of wheat and rice bran because it revealed features similar to fossil polyolefins encouraging to substitute them in several fields [18,19,20,21]. In fact the complementary nature of the two polymers (PLA is brittle with a slow crystallization rate [22,23], while PBSA, being ductile, shows a stiffness and mechanical strength that are not high [24,25]) offers a unique opportunity to capitalize their common characteristics [26].

Regarding the interfacial agent, a commercial beeswax has been used. Beeswax is a biobased wax constituted by a complex mixture (more than 300 components) of hydrocarbons, free fatty acids, esters of fatty acids and fatty alcohol, diesters and exogenous substances [27]. Beeswax is a beneficial option for polymer processing when polysaccharides are used because of its high hydrophobic nature and excellent water barrier properties [28]. In fact, Gigante et al. [29] published a work in which PHBV/bran bio-composites were processed with bioderived waxes by twin-screw extrusion to improve matrix-fiber compatibility. This approach was useful because, coating the fiber, the waxes reduce their hydrophilicity by increasing its surface roughness, thus improving the polymer adhesion to the matrix. In this framework, promising results have been obtained recently for different systems of natural fibers, that showed a better adhesion with polyester matrices in presence of beeswax [30,31,32]. This approach represents a good novelty in the field of matrix/fiber adhesion for bio-composite systems.

These bio-composites, after ensuring that adhesion at the interface has been improved, must also be further implemented in terms of mechanical resistance above room temperature. To achieve this goal, mineral fillers can be taken into account and added into the formulations. In fact inorganic additives have always been investigated in engineered polymers with the aim to guarantee a higher melt strength [33], to contain shrinkage and deformations [34], to increase the thermomechanical properties [35], to accelerate the crystallization rate in the molding time [36], to improve the anti-blocking properties (as natural fibers tend to bond under pressure and heat) [37] and, last but not least, decrease the final cost of the formulation itself [38]. The cheapest inorganic additives broadly available on the market, with the characteristics previously described, are talc and calcium carbonate [39]. Favorable effects of these fillers as nucleating agents in biopolymer packaging materials were evidenced in many literature works [40,41,42,43,44]. It was notable that a highly crystalline polymer has an HDT value higher than its amorphous counterpart [45] and it can be related to the stiffness of the resulting composites [46]. 

To be certain, the aim of the present paper, after the characterization of wheat bran and rice bran identified as forward-looking by-products, is the investigation of a preliminary beeswax-based treatment on these brans in order to improve their adhesion to the polymeric matrix of a PLA/PBSA blend. Moreover, two micrometric mineral fillers, talc and calcium carbonate, were examined by performing a well-rounded set of tests aimed at improving thermomechanical properties and heat deflection temperature of the obtained biocomposites.

## 2. Materials and Methods

### 2.1. Materials and Processing

- The polymeric matrix is a blend of biopolyesters constituted by 60 wt.% of PLA and 40 wt.% of PBSA, more specifically the PLA used was an extrusion grade PLA provided by Total Corbion: Luminy LX175. (D-lactic acid unit content 4%, density: 1240 kg/m^3^; melt flow index (MFI) (190 °C, 2.16 kg: 6 g/10 min). Conversely, PBSA was purchased from Mitsubishi Chemical Corporation with trade name BioPBS FD92PM. It is a ductile semicrystalline polyester, copolymer of succinic acid, adipic acid and butanediol (density: 1240 kg/m^3^; MFI (190 °C, 2.16 kg): 4 g/10 min).
- As natural fillers for the bio-composites formulations, Wheat Bran (WB) and Rice Bran (RB) residuals were used. They were provided by WeAreBio organic food; WB appears as light brown powder with hemicellulose content of 43 wt.% with bulk density of 250 kg/m^3^; RB looks, instead, dark yellow with hemicellulose content of 37 wt.% and bulk density of 320 kg/m^3^.
- With the aim to improve the matrix/natural filler adhesion and fillers dispersion, a non-ionic aqueous emulsion of beeswax (Aquacer T561, density of 980 kg/m^3^) purchased from BYK (Wesel, Germany) was exploited.
- Mineral fillers were also employed in a second phase of this work to investigate the variation of thermomechanical properties, to modify the melt strength, to optimize the formulation cost and to contain shrinkage during the molding. Calcium carbonate OMYACARB 1-AV purchased from Omya (Avenza/Carrara, Italy) and Talc Jetfine 0.7CA, supplied by Imerys (Roswell, GA, USA) were selected. CaCO_3_ showed a quasi-spherical shape with an average particle diameter of 1.6 µm; while talc, average dimensions lower than 1 µm, exhibited an ultra-lamellar aspect.

The treatment with the biobased wax of the wheat and rice bran fibers was conducted in a knife blender operating at 50 rpm, slowly injecting emulsion. Wheat bran and rice bran were dried in a stove for 2 days at 140 °C and then mixed with 4 wt.% and 8 wt.% of natural beeswax with respect to the bran content to achieve a narrower granulometric distribution. The idea was that thermally pre-treating bran fillers, sieving also for avoiding the fraction below 300 microns, eliminated the undesired effect of low adhesion wax/fiber. Finally, the sieved and selected WB and RB were kept in a stove at 90 °C for 24 h and packed hot in heat sealable bags before the following steps. 

A semi-industrial COMAC twin-screw extruder (Milan, Italy) equipped with two 25 mm co-rotating screws in a barrel with L/D = 44 was used to produce the bio-composites formulations for the evaluation of beeswax as interfacial agent and those with talc and calcium carbonate as thermomechanical properties enhancers. Operatively, PLA/PBSA pre-mixed granules were fed into the main hopper of the extruder, the inorganic filler (talc or calcium carbonate) was fed by a tailored lateral hopper for powders, at 2/3 of the screws length, while, the bran, once grinded, covered (or not) by the wax and then dried, was inserted by another side feeder at 3/4 of the screw length. The temperature profile adopted for all the composites was 150/170/180/180/185/185/190/190/185/185/185 °C. The screws rate was kept at 300 rpm and the mass feed at 15 kg/h. The filaments at the outlet of the nozzle were continuously cooled in a tank of water, dried by a constant jet of air and then granulated in a mechanical cutter obtaining pellets with a diameter of 1.5 mm. 

All the pellets were finally dried for 8 h in a PIOVAN DP 604–615 dryer at 50 °C and then, utilized to obtain ISO 527 1-A dog bone samples (for tensile tests) and ISO 179 parallelepiped shape specimens (for Charpy and HDT) by an injection molding machine (Megatech H18/10, Fabriano (AN), Italy). The temperature profile used was the following: 175/180/185 °C and the mold was kept at 60 °C for 10 s with an injection pressure of 120 bar. The composition (wt.%) of the developed bio-composites is reported in Table 1. 

### 2.2. Testing Methodologies

Thermogravimetric analysis (TGA) was accomplished using a TA Q-500 (TA Instruments, Waters LLC, New Castle, DE, USA). Around 15 mg of the specimen to be tested has been inserted into a platinum pan and heated from 25 °C to 600 °C at 10 °C/min under nitrogen atmosphere.

The morphological features of wetted (and not) bran fibers, the length distribution and the matrix/filler adhesion, were analyzed by scanning electron microscope (SEM) FEI Quanta 450 FEG (ThermoFisher Scientific, Waltham, MA, USA) equipped with a large field detector for low kV imaging simultaneous secondary electron (SE). The powders were prior sputtered with a layer of platinum that makes the surface electrically conductive. The WB and RB dimensional was calculated from the SEM images obtained using the Image J^®^ software(NIH, Bethesda, MD, USA). About 200 particles were examined for each powder typology.

Melt flow rate (MFR), (according to the ISO 1133:2005) was also evaluated through a CEAST MF20 (Canton, MA, USA) to evidence the melt strength differences. The temperature selected for the tests was 190 °C A pre-heating time of 30 s followed by the application of a 2.16 kg weight were selected.

Quasi-static tensile tests have been performed at 25 °C with an MTS Criterion 43 universal testing machine, equipped with a 1 kN load cell and interfaced with a computer running Test Suite 4.0. The specimen used were molded following ISO 527 1-A specimens. 10 mm/min of crosshead speed has been selected. At least five specimens for each formulation were tested and the average values were reported. 

Impact tests were carried out using an Instron CEAST 9050 (Canton, MA, USA) and ISO 179 V-notched specimens (V-notch 2 mm at 45°) at 25 °C. The standard method ISO179:2000 was followed. For each formulation, at least five specimens were tested, and the average values were reported. 

HDT A test (which assesses at what temperature a specimen 80 mm × 10 mm × 4 mm subjected to three-point bending deflected of 0.34 mm under a pressure of 0.45 MPa) were also performed on a HVT302B (MP Strumenti, Italia) in accordance with ISO 75-1 (method A).

In order to identify the crystallization behavior of the bio-composites of the two different phases, thermal properties were investigated by calorimetric analysis (Q200 TA Instrument, Waters LLC, New Castle, DE, USA). The method set to perform the thermal analysis (considering only a single heating ramp to evaluate the crystallinity linking it with the manufacturing process) was:

- Jump up to −50 °C and kept at this temperature for 1 min;
- Ramp up to 200 °C, at 10 °C/min and isotherm for 1 min at 200 °C;
- Jump up to 25 °C.

By using the TA Universal Analysis software, it is possible to determine glass transition temperature (Tg), melting temperature (Tm), cold crystallization temperature (Tcc), cold crystallization enthalpy (ΔHcc) and melting enthalpy (∆Hm). In addition, the percentage of crystallinity of PLA (Xc) can be obtained through the Equation (1):

(1) $$X_{C,\mathrm{PLA}}=\frac{{\Delta H}_{m,\mathrm{PLA}}-{\Delta H}_{\mathrm{cc},\mathrm{PLA}}}{{\Delta H}_{m,\mathrm{PLA}}^{0}\cdot \mathrm{wt}}$$

where ∆H_m,PLA_ and ∆H_cc,PLA_ are the melting enthalpy and the enthalpy of cold crystallization of PLA obtained in J/g, wt is the weight fraction of PLA that crystallizes and ∆H^0^_m,PLA_ is the melting enthalpy of the 100% crystalline PLA, equal to 93 J/g [47].

## 3. Results and Discussions

### 3.1. Evaluation of Natural Filler Features and Influence of Beeswax

#### 3.1.1. Thermogravimetric Analysis (TGA)

**Figure 1:**
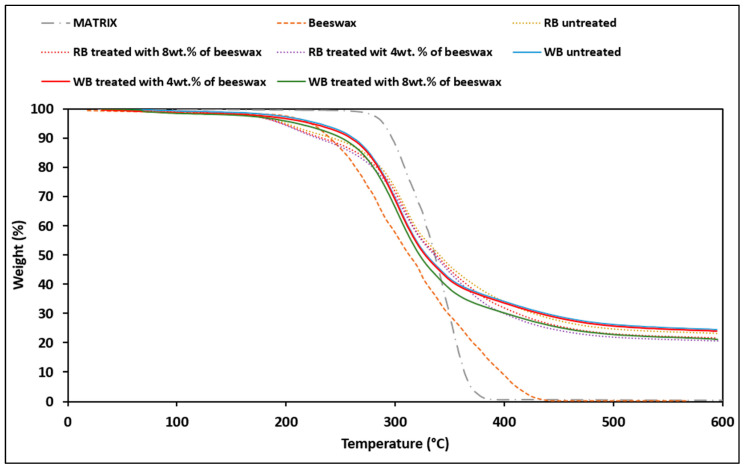
TGA of beeswax, PLA/PBSA matrix, Wheat Bran and Rice Bran with and without different percentage of beeswax treatment.

The first information given by the TGA analysis (Figure 1) on natural bran (WB and RB), with and without beeswax, comparing them with beeswax as it is and the thermogravimetric behavior of PLA/PBSA matrix, is the effectiveness of the drying process since up to 100 °C there are no mass losses due to evaporation of water. The behavior of the wheat bran samples is similar with and without beeswax treatment, indeed it starts to degrade not lower than 230 °C. Wax tends to cause higher weight losses at slightly lower temperatures but, within an acceptable range, there are no obvious differences in behavior as the temperature increases. Slightly different is the behavior of the rice bran, in fact Figure 1 shows two different changes of slope, a first lighter one around 185 °C and a more evident one at 210 °C with evident loss of weight until 400 °C where the weight stabilizes as many other natural fillers [48].

The biobased wax shows its degradation in the range 200–390 °C, so it is stable at the temperatures used in classical processing operations, as the PLA/PBSA blend used as matrix. In any case, both residues and matrix can be considered stable and suitable for further processing (extrusion and injection molding).

#### 3.1.2. Morphological SEM Analysis of the Treated and Untreated Fibers

**Figure 2:**
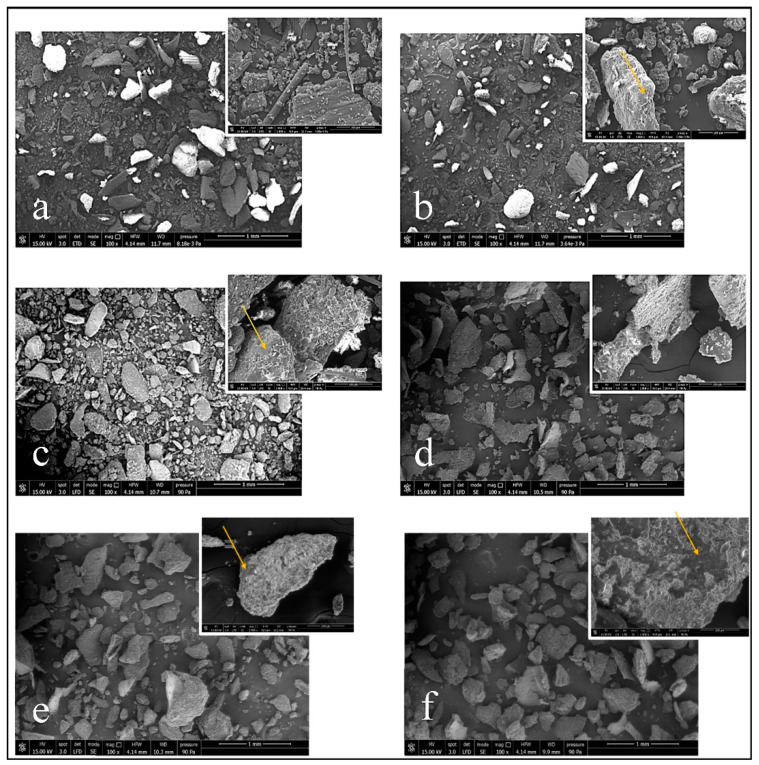
SEM of (**a**) Wheat (**b**) WB + 4 wt.% beeswax (**c**) WB + 8 wt.% beeswax (**d**) Rice (**e**) RB + 4 wt.% beeswax (**f**) RB + 8wt.% beeswax. Yellow arrows to highlights the wax wetting.

To investigate the microstructure and the dimensional distribution of WB and RB, the powders were analyzed by Scanning Electron Microscope (SEM). The WB and RB with a magnification of each typology is reported in Figure 2. It can be observed that both WB and RB powders consist of platelets having low aspect ratio with a tendency to form agglomerates. The surface morphology of wheat bran displayed the incidence of protein, starch, fat, and globular particles as already evidenced in literature [49]. The presence of a smooth surface over bran leaf, called cuticle, identified as aliphatic wax [50], was evident. Zooming in deeper on the flakes (both wheat and rice) treated with 4 and 8 wt.% of beeswax, it is possible to see how the emulsion has been deposited on the particles surface (Figure 2b,c,e,f). 

To calculate the mean aspect ratio of both powders, the flake geometry must be considered for which the aspect ratio is defined as the ratio between the average fiber diameter (*d*) (calculated on the larger platelets surface) and the mean thickness of the platelets (*h*) according to the following equation [51] (Equation (2)):

(2) $$a_{r},platelets=\frac{d}{h}$$

**Figure 3:**
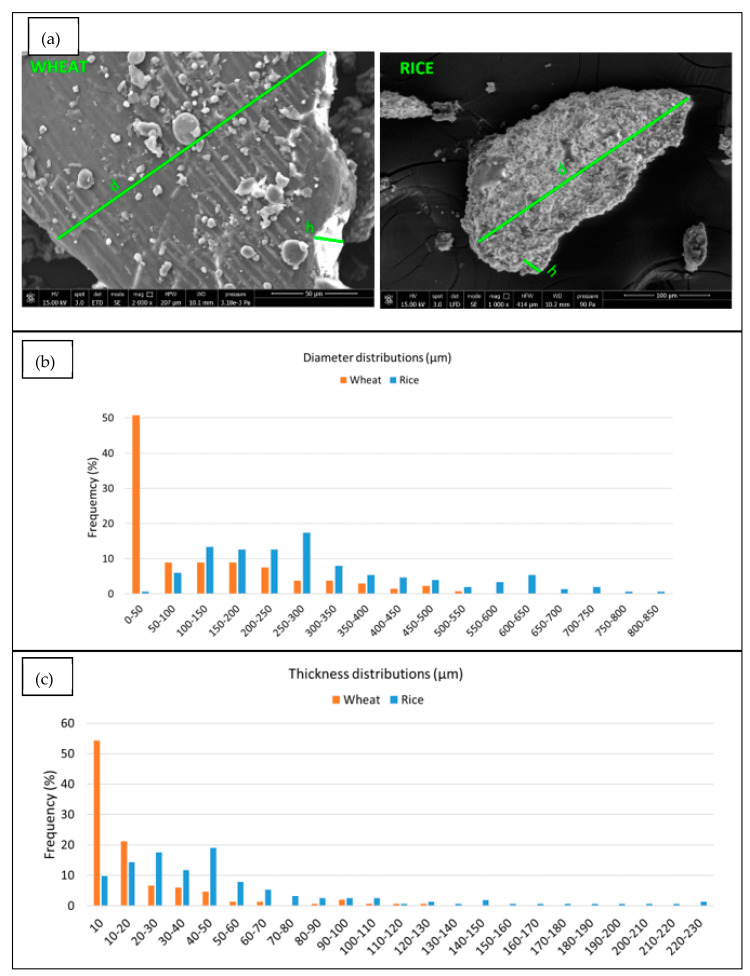
(**a**) 2000× magnification of Wheat Bran and Rice Bran (**b**) diameter distribution (**c**) thickness distribution.

In Figure 3a it is possible to observe how d and h parameters were calculated through the Image J software and the results of the dimensional distributions are reported in Figure 3b,c. It can be observed that the particle size distribution of WB (both in terms of thickness and diameter) is narrower with a smaller average particle size respect to RB. Thus, the surface area of WB is reasonably higher than RB. On the other hand, RB has a wide-ranging sizes distribution. Nevertheless, the average mean weighted aspect ratio is comparable between the two powders with a value of 5.29 and 4.22 for WB and RB, respectively.

### 3.2. Melt Fluidity

**Figure 4:**
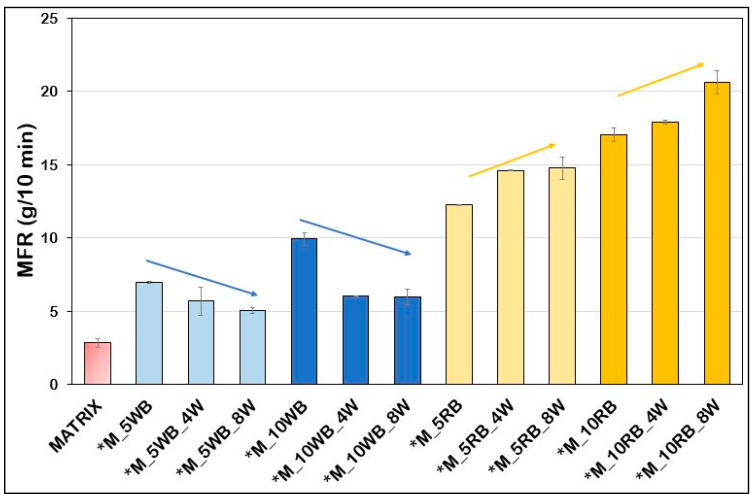
Melt flow rate behavior for the formulations with beeswax, showing opposite trends.

**Figure 5:**
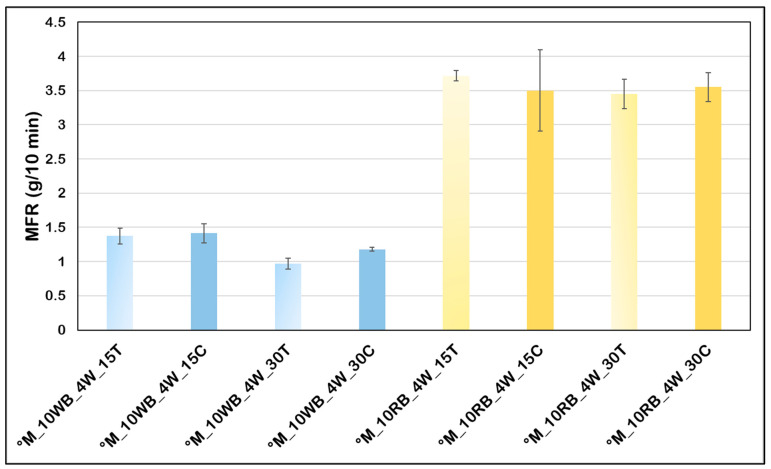
Melt flow rate behavior inserting 10 wt.% of coated fibers with different amounts of particulate mineral fillers.

The bran effect on the bio-composite fluidity index was investigated by the Melt Flow Rate (MFR) measurements both for bio-composites formulations for the evaluation of beeswax as interfacial agent and those with talc and calcium carbonate as thermomechanical properties enhancers (Figure 4 and Figure 5). Starting from Figure 4, it can be noticed that the lowest value of MFI is the one of the pure matrix; indeed, the addition of wheat bran and, above all, rice bran determined a strong increase in MFR. This increase can be ascribed at a reduction of molecular weight through chain scission with the consequent decrease in melt viscosity. Despite the bran fibers drying pretreatment, some residual moisture probably remained, causing the melt flow value to rise [20]. Similar results were achieved by Aliotta et al. [52], Gorrasi et al. [53], Gigante et al. [54] and, above all, by Barczewski et al. [55] in which the MFI value grows along with increasing chestnut content. However, an interesting fact to underline is that the beeswax, while lowering the fluidity index of wheat bran fibers-based composites, caused a considerable MFR increase in the case of rice bran fibers composites, suggesting a possible plasticizing effect of this additive. 

Regarding the composition of wheat and rice bran it is well known that the latter has a concentration of lipids, mainly consisting in triacylglycerols, higher. In particular, the content of lipids in rice bran is between 15 and 20 wt.% [56], whereas in wheat bran the lipids do not exceed 5.6 wt.% [57]. As lipids are liquid in the condition of processing, this compositional difference explains the higher MFR of Rice bran composites. Moreover, the addition of wax determines only a plasticization in the case of rice bran (with the consequent increase in fluidity). On the contrary, despite of its plasticizing action, the wax added to the wheat bran composite determines a slight but significant decrease in MFR, reasonably in agreement with the arise of new interactions between the different components of the composites. In particular, an increased compatibility between the coated wheat bran and the apolar biopolyesters matrix can be reasonably hypothesized.

Through the analysis of melt fluidity for bio-composites with bran and rice “wax-coated” but with the addition of talc or calcium carbonate as thermomechanical properties enhancers (Figure 5). Firstly, it can be noticed that all the resulted values are much lower than in the graph of Figure 2 demonstrating that, maintaining constant the operative temperature, the inclusion of an organic filler guarantees an increase in melt strength. Nevertheless, the tendency to higher MFR values for bio-composites with rice bran is confirmed, thus showing a greater melt fluidity increase effect in the long term, but compared to bio-composites without particle fillers, the values are 4/5 times lower. In particular, the addition of 30 wt.% of talc to the bio-composite which already had 10 wt.% of wheat bran allows to reach a melt flow rate below 1 g/10 min. On the other hand, the values of RB compounds are about 3.5 g/10 min both with talc or carbonate, and both with 15 wt.% or 30 wt.% of mineral filler.

#### 3.2.1. Mechanical Properties

In the evaluation of the quasi-static tensile tests results for the evaluation of beeswax as interfacial agent are showed in Table 2 where the experimental work has been compared with literature mechanical values of bran-based bio-composites developed for rigid applications, displaying how they are similar to the results of the present paper. More specifically, it is evident how the behavior of melt fluidity is reflected in the trend of RB mixtures: in fact, a lowering of the elastic modulus is evident with the increase of the quantity of RB as stated also by Nwosu et al. [58]. On the contrary, this behavior is not present for the bio-composites with wheat bran with and without wax in which the Young’s Modulus remains almost constant also with respect to the value of the matrix, showing a trend similar to MFR. Compared to the matrix, instead, the elongation at break collapses as was to be expected given the size of the flakes of both bran and rice: they act as stress intensity factors and defect points from which the propagation of the crack starts [59].

There are no substantial differences also with regard to the composite impact resistance, indeed the Charpy Impact Strength is about 4 kJ/m^2^ for bio-composites with WB and about 5 kJ/m^2^ for bio-composites with RB. However, it seems that the treatment with 4% wax guarantees higher values of impact resistance. This assumption is also confirmed by the higher stress at break values both for WB and RB at 4 wt.%, and all the stress at break values (increasing the content of fillers) are very similar to the matrix resistance evidencing, therefore, a good matrix/filler adhesion. This is a good point, indeed usually a reduction of the break strength with natural filler content is observed [61,62], due to a poor interfacial interaction and consequent inefficient load transfer between polymeric matrix and filler. 

The outcome that the use of 4% of beeswax guarantees better results than a higher quantity is to be found in the type of action that the additive plays. Probably, by increasing its concentration, beeswax not only acts as an interfacial agent but, slightly, also as a plasticizer, thus lowering the values of stress at break and, above all, of stiffness and at the same time increasing the elongation at break.

In order to confirm this good adhesion and to analyze in detail if the “4% wax mixtures” could be the best formulations both in the case of WB and RB, an analytical model has been applied through the calculation of the Pukanszky *B* factor [63]. 

Starting from *B* factor, in Pukanszky’s model the reinforcing effect of filler is expressed quantitatively by considering the effect of the decrease in effective load-bearing cross-section of the polymer (Equation (3)):

(3) $$\ln\sigma_{c,red}=\ln\frac{\sigma_{c}\left(1+2.5\;V_{p}\right)}{1-V_{p}}=\ln\sigma_{m}+BV_{p}$$

where $\sigma_{c,red}\;$ is the reduced tensile strength, i.e., the tensile strength normalized to the cross-section perpendicular to the load direction, $\sigma_{c}$ and $\sigma_{m}$ are the break stress of the composite and the matrix, respectively, *V_p_* is the filler volume fraction and *B* is a parameter connected to the matrix/filler interaction. From the slope of the logarithm of $\sigma_{c,red}$ against *V_p_*, the value of the *B* parameter can be determined. 

*B* has no direct physical meaning, but it relates to the interfacial properties of the system. In a simplified way the higher is *B* the better is the adhesion.

**Figure 6:**
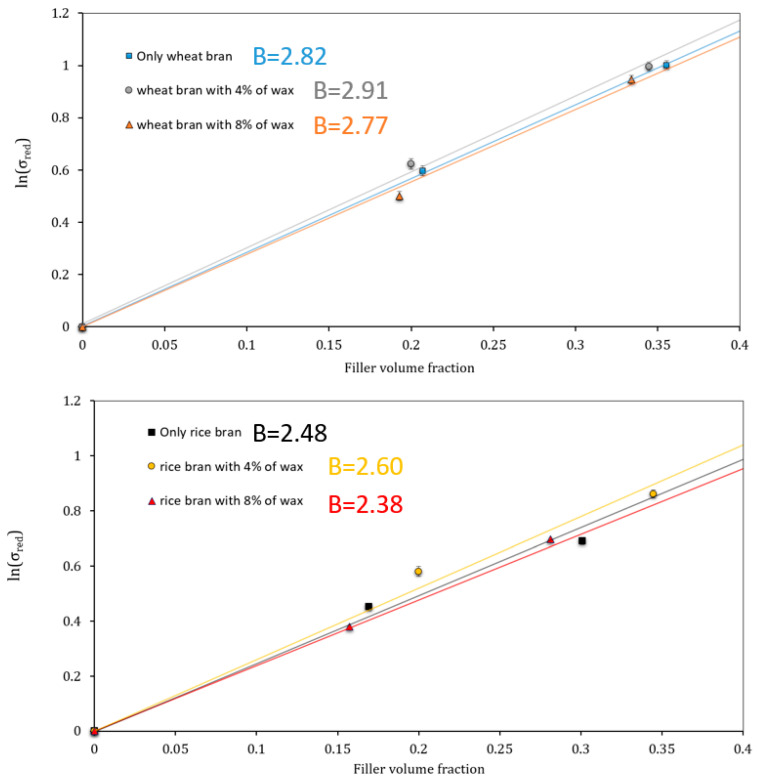
Pukanszky’s analytical model application that demonstrates the better adhesion matrix/filler with a 4% wax treatment.

The results are highlighted in Figure 5. As shown, the treatment of bran filler with 4 wt.% of wax ensured better adhesion than that of the other two series of composites with WB and RB because the slope of the trend line (which represents the Pukanszky’s *B* parameter, Figure 6) is, even slightly, higher with a slight predominant adhesion effect in the system wheat bran/PLA-PBSA. 

The effectiveness of a beeswax-based treatment useful to increase the adhesion and dispersion of WB and RB fibers in the polymer matrix was investigated. Based on the results, 10% fiber bio-composites (excellent compromise between the amount of natural fiber and mechanical properties) treated with a 4% beeswax wetting were selected. 

A particulate mineral filler can be suitable for the improvement of thermos-mechanical properties of the Bio-composites, ensuring also higher melt strength to increase melt viscosity, thus counterbalancing the fluidity enhancing effect of bran. 

Regarding Table 3, that shows the tensile and impact mechanical properties of the mineral filled bio-composites, it is evident that the compounds °M_10WB_4W_30T and °M_10RB_4W_30T showed a significantly higher stiffness (above 4 GPa) than the other bio-composites due to the effective talc reinforcing efficiency and the highest filler content.

This trend can be also evidenced even if 15% of talc is added, because there is a slight increase in young’s modulus with respect to unreinforced bio-composites (Table 2). This data is reflected in stress resistance behavior that, both for materials with WB and RB, were greater in the presence of talc (the opposite effect, however, is the collapse of the elongation value at break, which is irrelevant if the final application should be a rigid and thermomechanically resistant item). The addition of calcium carbonate, on the other hand, did not increase the elastic modulus, the values around 2 GPa. The calcium carbonate probably acts only as a slip agent [64] promoting an increase in tensile toughness and higher ductility values. A very interesting behavior is connected the impact properties. 

Despite the addition of a large amount of mineral filler that could have embrittled the final bio-composite, the Charpy Impact Strength values are higher than those of unreinforced ones and it can be stated that a process of rigid filler toughening [65] has occurred in which the voids around the particle filler, as stated by Argon and Cohen theory [66], have acted as an obstacle to crack propagation.

Definitely the investigation of bio-composites mechanical properties showed that high stiffness and tensile strength, connected with low elongation at break, is a prerogative of wheat bran-based bio-composites. Rice bran fibers, instead, cause a lowering of the modulus but an improvement in elongation at break, reflecting the trend of melt strength. The values of Charpy Impact Strength showed values near 5.5 kJ/m^2^ for all the bio-composites.

#### 3.2.2. Heat Deflection Temperature Measurements 

A property that may be discriminating in the use of one bio-composite over another may also be its ability to resist to deformation at temperatures higher than room temperature. For example, in the field of tableware, the contact with hot food and beverages could deflect and deform items that, instead, at room temperature have very good mechanical properties. Precisely, in order to improve the thermomechanical resistance, the use of talc and calcium carbonate assumes fundamental importance, indeed the use of an organic filler, could ensure an increase in stiffness above the glass transition that would be reflected in a greater tendency to resist to deformation by increasing the temperature [67].

**Figure 7:**
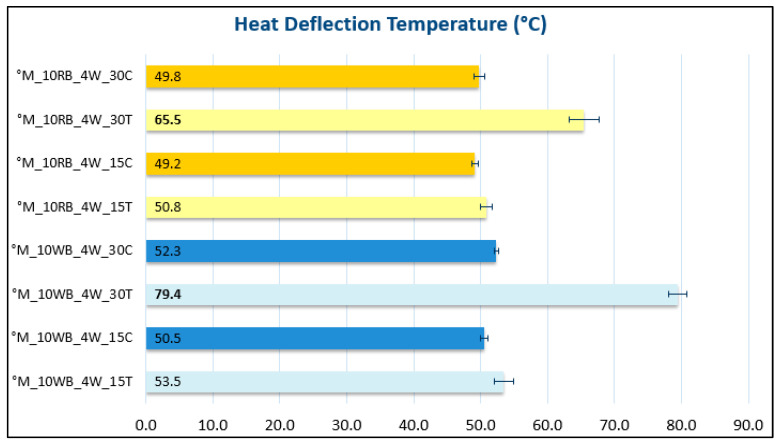
HDT measurements of inorganic filled biocomposites.

The results of the HDT characterization shown in Figure 7 highlight an eloquent result: the addition of calcium carbonate in any amount or talc in small quantities resulted in a maximum HDT temperature at about 50 °C. However, the introduction of 30 wt.% of talc in the bio-compounds with 10 wt.% of wax-wetted RB and WB allowed the achievement of a much higher resistance to thermal deflection, respectively, up to 65 °C and even up to 80 °C. This result is in agreement with the results demonstrated by Phetwarotai et al. [68] in which polylactide acid based films were nucleated with talc or calcium carbonate the bio-composite were prepared in a twin-screw extruder. The results showed that the tensile properties, thermal stability, spherulitic morphology and PLA crystallization behaviors of the nucleated PLA blends depended significantly on the quantity of talc and calcium carbonate; moreover Lee et al. [69] stated that increasing the quantity of talc, the thermal resistance of PLA can be improved but at the cost of lower tensile strength and elongation at break.

#### 3.2.3. Crystallinity Evaluation through DSC Analysis

**Figure 8:**
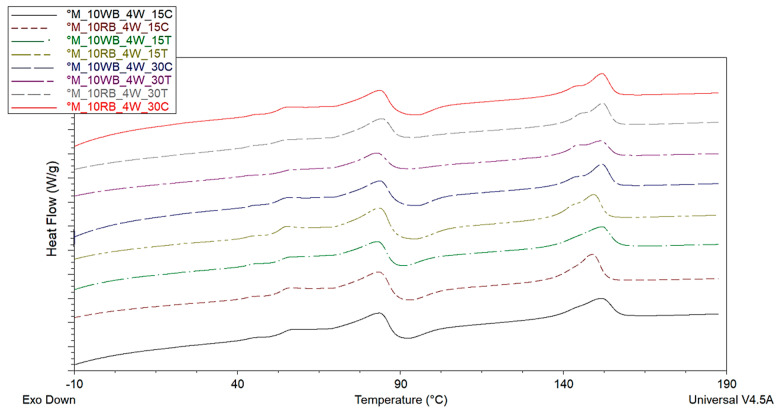
DSC analysis of mineral reinforced biocomposites.

To identify the crystallization behavior of the bio-composites connecting it to the HDT results, considering only the first heating to evaluate the crystallinity due to the manufacturing process, DSC characterization was carried out (Figure 8).

The results concerning the percentage of crystallinity (Table 4) showed that only formulations in which approximately 30% crystallinity is achieved are those which show the highest HDT values. This is caused by low values of the ΔHcc value, so the addition of a high quantity of talc, in connection with the increase in the mold temperature and the cooling time, allowed the material to crystallize during cooling, thus avoiding an extensive cold crystallization.

Talc resulted more efficient that calcium carbonate in inducing an increase in crystallinity. The layered structure typical of talc is very efficient in polyester nucleation with respect to the pseudo-spherical particles of calcium carbonate [70]. Hence, talc showed not only a significant reinforcing action, but also a determinant influence on crystallinity development that reached a content in the matrix of about 30%. In these specific cases (°M_10WB_4W_30T and °M_10RB_4W_30T), both the crystalline phase (melting at about 150°C), the bran and the filler mineral phase do not deform above the matrix glass transition, accounting at about 70 wt% of the composites. Hence, the whole composite becomes significantly more resistant to heat distortion above PLA glass transition.

#### 3.2.4. Morphological Aspect of Cryo-Fractured Surfaces

**Figure 9:**
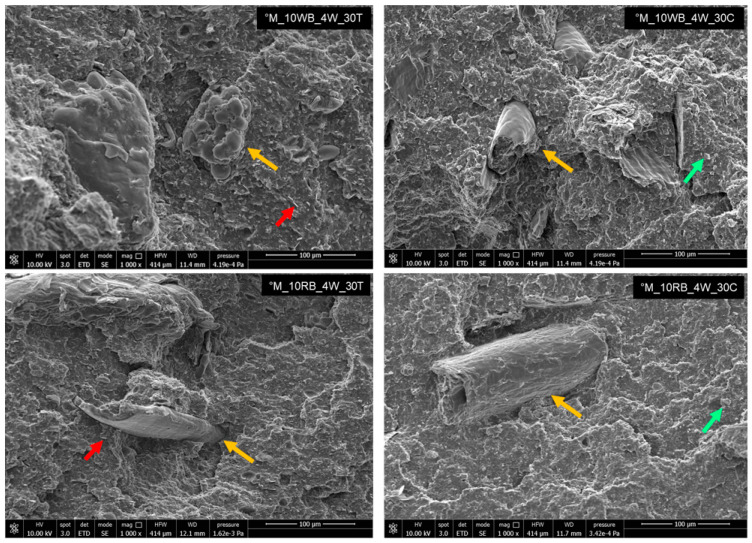
SEM micrographs with 1000× of magnification of optimized biocomposites with WB (**up**) and RB (**down**) with 30 wt.% of talc (**on the left**) and 30 wt.% of calcium carbonate (**on the right**).

The morphological study of the composites with 30% mineral fillers for both treated agricultural residues evidenced the complexity of the obtained composites because of the presence of different phases (Figure 9). However, this analysis showed that the treatment with 4 wt.% of beeswax guaranteed an excellent filler matrix adhesion (yellow arrows).

The red arrows highlight the lamellar nature of talc, which, despite being in very high quantity, is well distributed inside the PLA-PBSA matrix. Moreover, the uniform dispersion of the quasi-spherical calcium carbonate particles (shown in Figure 9 with the green arrow) is evident. Consequently, the morphological features, seem in agreement with interaction parameter results and thermomechanical considerations. 

## 4. Conclusions

The present work had the objective to improve the interfacial adhesion and the thermomechanical properties of a peculiar bio-composite system constituted by a blend of PLA and PBSA used as matrix and platelets of wheat and rice bran dispersed as fillers, valorizing and reusing them being an agricultural byproduct. Wheat bran and rice bran have been morphologically characterized (in terms of shape, aspect ratio and dimensional distribution) and pre-coated with a beeswax treatment to improve the compatibility with the polymeric matrix. It has been demonstrated that beeswax promoted filler/matrix adhesion, but it also acts as plasticizer for the composite. An optimum value of 4 wt.% of beeswax was selected for bran pre-treatment. 

Moreover, to improve the thermo-mechanical properties of the bio-composites, the introduction of mineral fillers such as talc and calcium carbonate was investigated registering a significant reduction in melt fluidity. In the paper it has been shown that 30 wt.% amount of talc affected the kinetics of crystallization of PLA resulting in the highest crystallinity value, thus ensuring to the bio-composite a greater resistance to thermal deflection. Interestingly, the reinforcement of a bio-polyesters matrix both with an agricultural and mineral filler were integrated to reach this objective.

As the thermal distortion is considered a weak point in green composites based on PLA, these results can be significant for developing fully biobased materials with a reduced heat distortion, thus allowing more and more applications of these environmentally friendly materials for rigid applications, ranging from food packaging to automotive up to electric/electronic devices. 

## Figures and Tables

**Table 1:** Mass composition (wt.%) of studied bio-composites.

Acronym	PLA	PBSA	Wheat Bran (WB)	Rice Bran (RB)	Beeswax (W)	Talc (T)	Calcium Carbonate (C)
MATRIX	60	40	/	/	/	/	/
*M_5WB	57	38	5	/	/	/	/
*M_5WB_4W	57	38	4.8	/	0.2	/	/
*M_5WB_8W	57	38	4.6	/	0.4	/	/
*M_10WB	54	36	10	/	/	/	/
*M_10WB_4W	54	36	9.6	/	0.4	/	/
*M_10WB_8W	54	36	9.2	/	0.8	/	/
*M_5RB	57	38	/	5	/	/	/
*M_5RB_4W	57	38	/	4.8	0.2	/	/
*M_5RB_8W	57	38	/	4.6	0.4	/	/
*M_10RB	54	36	/	10	/	/	/
*M_10RB_4W	54	36	/	9.6	0.4	/	/
*M_10RB_8W	54	36	/	9.2	0.8	/	/
°M_10WB_4W_15T	45	30	9.6	/	0.4	15	/
°M_10RB_4W_15T	45	30	/	9.6	0.4	15	/
°M_10WB_4W_15C	45	30	9.6	/	0.4	/	15
°M_10RB_4W_15C	45	30	/	9.6	0.4	/	15
°M_10WB_4W_30T	36	24	9.6	/	0.4	30	/
°M_10RB_4W_30T	36	24	/	9.6	0.4	30	/
°M_10WB_4W_30C	36	24	9.6	/	0.4	/	30
°M_10RB_4W_30C	36	24	/	9.6	0.4	/	30

**Table 2:** Quasi-static mechanical properties and impact behavior of studied bio-composites.

Acronym	Young’s Modulus (GPa)	Stress at Break (MPa)	Elongation at Break (%)	Charpy Impact Strength (kJ/m^2^)
MATRIX	1.99 ± 0.12	21.5 ± 0.8	192.8 ± 56.6	9.2 ± 0.5
*M_5WB	1.97 ± 0.07	20.4 ± 0.5	6.7 ± 0.1	4.0 ± 0.3
*M_5WB_4W	2.02 ± 0.02	21.4 ± 0.7	5.3 ± 0.2	4.0 ± 0.2
*M_5WB_8W	2.01 ± 0.12	19.3 ± 0.3	6.0 ± 0.2	3.8 ± 0.1
*M_10WB	2.06 ± 0.04	20.0 ± 1.3	4.3 ± 0.3	3.8 ± 0.1
*M_10WB_4W	2.03 ± 0.13	20.8 ± 0.9	3.6 ± 0.3	4.0 ± 0.2
*M_10WB_8W	2.00 ± 0.08	20.1 ± 0.3	4.0 ± 0.4	3.9 ± 0.3
*M_5RB	1.79 ± 0.13	19.7 ± 1.4	10.9 ± 2.7	5.0 ± 0.2
*M_5RB_4W	1.81 ± 0.06	20.5 ± 0.5	11.4 ± 1.2	5.6 ± 0.8
*M_5RB_8W	1.73 ± 0.02	19.0 ± 0.2	11.8 ± 2.5	5.0 ± 0.3
*M_10RB	1.69 ± 0.14	17.1 ± 0.1	6.2 ± 0.8	4.8 ± 0.2
*M_10RB_4W	1.48 ± 0.02	17.9 ± 0.2	6.1 ± 0.2	4.8 ± 0.6
*M_10RB_8W	1.40 ± 0.10	18.2 ± 0.8	6.2 ± 0.7	4.8 ± 0.1
PBS+ 50 wt. % wheat bran [60]	1.6 ± 0.09	13.5 ± 0.1	8.1 ± 0.9	8.9 ± 0.7
PHBV + 10% wheat bran [29]	2.1 ± 0.10	18.9 ± 0.2	2.0 ± 0.1	3.8 ± 0.2
PP + 30% wheat bran [10]	2.30 ± 0.10	19.9 ± 0.3	6.2 ± 0.4	4.1 ± 0.2

**Table 3:** Quasi-static mechanical properties and impact behavior of bio-composites with inorganic fillers.

Acronym	Young’s Modulus (Gpa)	Stress at Break (MPa)	Elongation at Break (%)	Charpy Impact Strength (kJ/m^2^)
°M_10WB_4W_15T	2.89 ± 0.09	24.1 ± 1.0	4.9 ± 0.1	5.4 ± 0.5
°M_10WB_4W_15C	2.21 ± 0.06	21.5 ± 0.1	4.1 ± 0.6	4.7 ± 0.4
°M_10WB_4W_30T	4.50 ± 0.32	28.2 ± 1.6	1.5 ± 0.1	5.5 ± 0.7
°M_10WB_4W_30C	2.94 ± 0.14	20.6 ± 0.9	2.3 ± 0.2	5.4 ± 0.8
°M_10RB_4W_15T	2.38 ± 0.17	20.3 ± 1.0	9.1 ± 0.9	5.2 ± 0.9
°M_10RB_4W_15C	2.02 ± 0.05	17.3 ± 0.7	11.3 ± 2.0	5.1 ± 1.1
°M_10RB_4W_30T	4.21 ± 0.10	23.2 ± 0.3	1.9 ± 0.2	5.3 ± 0.8
°M_10RB_4W_30C	2.08 ± 0.09	18.5 ± 0.9	5.5 ± 0.7	5.1 ± 0.8

**Table 4:** Results of differential scanning calorimetry (DSC) analysis (first heating).

Acronym	Tcc (°C) PLA	Tm (°C) PLA	Tm (°C) PBSA	ΔHm (J/g) PLA	ΔHm (J/g) PBSA	ΔHcc (J/g) PLA	Xc (%) PLA
°M_10WB_4W_15T	92.1	151.4	83.1	14.7	8.1	6.0	22.3
°M_10WB_4W_15C	93.5	151.3	83.1	13.1	10.1	7.2	15.1
°M_10WB_4W_30T	93.1	150.7	82.8	10.9	6.4	1.6	**30.3**
°M_10WB_4W_30C	95.6	151.7	83.6	11.1	6.8	4.1	22.8
°M_10RB_4W_15T	92.5	148.7	83.4	14.1	7.3	5.8	21.2
°M_10RB_4W_15C	94.0	148.8	83.1	14.4	9.5	6.2	21.0
°M_10RB_4W_30T	92.5	151.9	84.0	10.7	8.3	1.7	**29.3**
°M_10RB_4W_30C	95.7	152.1	84.2	10.7	7.0	4.9	18.9

## Data Availability

Not applicable.
